# Supplementary material for: Impact of the First COVID-19 Wave on French Hospitalizations for Myocardial Infarction and Stroke: A Retrospective Cohort Study
Source: Biomedicines. 2022 Oct 7;10(10):2501. doi: 10.3390/biomedicines10102501 (PMC9598815; doi:10.3390/biomedicines10102501)

Supplementary file

Impact of the first COVID-19 wave on French hospitalizations for Myocardial infarction and Stroke: a retrospective cohort study

Anne-Sophie Mariet[1.2]. Gauthier Duloquin[3.4]. Eric Benzenine[1]. Adrien Roussot[1]. Thibaut Pommier[5]. Jean-Christophe Eicher[5]. Laura Baptiste[3.4]. Maurice Giroud[3.4]. Yves Cottin[5.6.7]. Yannick Béjot[3.4]. Catherine Quantin[1.2.8]

1. Biostatistics and Bioinformatics (DIM). University Hospital of Dijon. France
2. CIC1432. University Hospital of Dijon. Clinical Investigation Center. Clinical Epidemiology/Clinical Trials Unit. Dijon. France
3. Neurology Department. University Hospital of Dijon. France
4. Dijon Stroke Registry (Santé Publique France – Inserm) - EA 7460 (Pathophysiology and Epidemiology of Cerebro-CardioVascular Diseases). University of Burgundy. UFBC. Dijon. France
5. Cardiology Department. University Hospital of Dijon. France
6. Pathophysiology and Epidemiology of Cerebro-CardioVascular Diseases. University of Burgundy. Dijon. France
7. Registre des Infarctus du Myocarde de Côte d'Or. University Hospital of Dijon. France
8. Université Paris-Saclay. UVSQ. University of Paris-Sud. Inserm. High-Dimensional Biostatistics for Drug Safety and Genomics. CESP. Villejuif. France

Supplementary Figure S1. Trends of hospital admissions for myocardial infarction and its subtypes including cases associated with COVID-19 in January to September 2020 compared to 2019

Supplementary Figure S2. Trends of hospital admissions for cerebrovascular events and its subtypes including cases associated with COVID-19 in January to September 2020 compared to 2019

Supplementary Table S1. Crude and standardized rates of hospitalizations for cerebro-cardiovascular event in France in 2020 before. during and after the first lockdown

Supplementary Table S2. Interrupted time series analyses of hospitalizations for cerebro-cardiovascular events for weeks 2 to 39 of 2020 in France. with three periods: before. during and after the lockdown (weeks 12 to 19)

Supplementary Figure S3. Observed (Obs) and predicted (ITS) hospitalizations for cardiovascular events for weeks 2 to 39 of 2020 in France. interrupted time series (ITS) analysis with three periods (before. during. after the first lockdown)

Supplementary Figure S4. Observed (Obs) and predicted (ITS) hospitalizations for cerebrovascular events for weeks 2 to 39 of 2020 in France. interrupted time series (ITS) analyses with three periods (before. during. after the first lockdown)

Supplementary Figure S1. Trends of hospital admissions for myocardial infarction and its subtypes including cases associated with COVID-19 in January to September 2020 compared to 2019

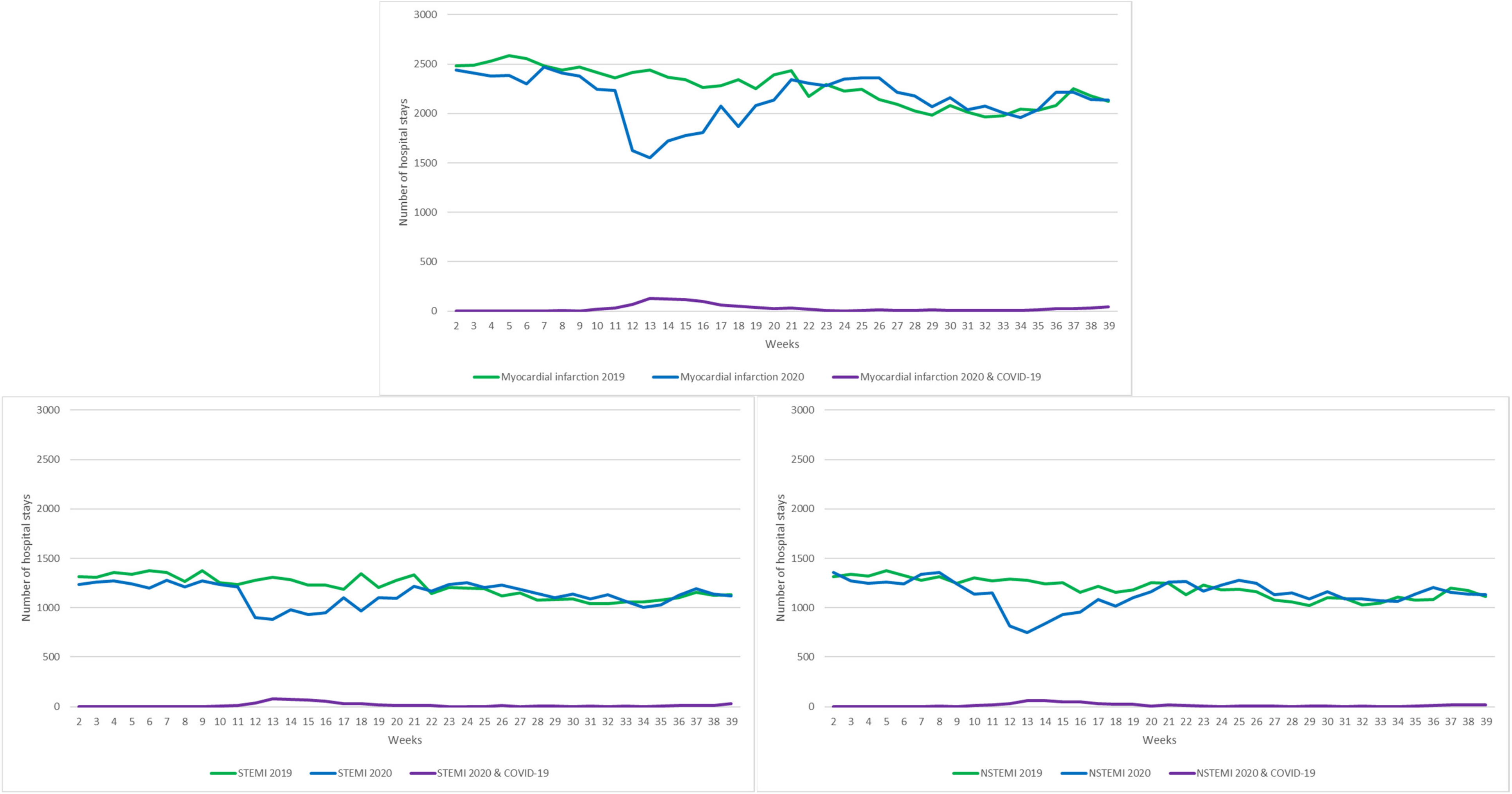

Supplementary Figure S2. Trends of hospital admissions for cerebrovascular events and its subtypes including cases associated with COVID-19 in January to September 2020 compared to 2019

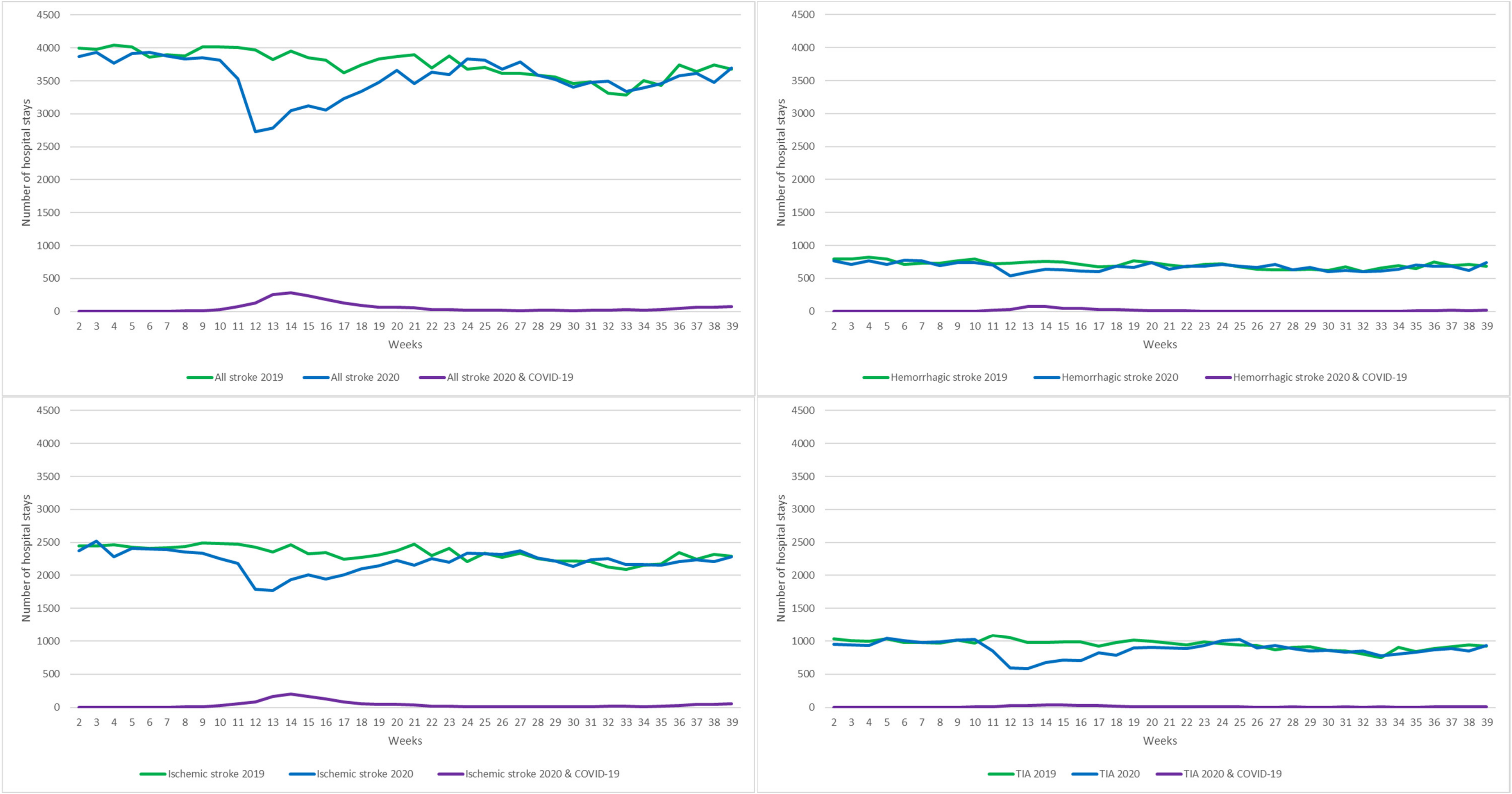

Supplementary Table S1. Crude and standardized rates of hospitalizations for cerebro-cardiovascular event in France in 2020 before. during and after the first lockdown

Hospitalizations for:	Crude rate (per 100.000 persons-months)			Standardized rate on European population (per 100.000 persons-months)			Standardized rate on world population (per 100.000 persons-months)		
	Before[a]	During[b]	After[c]	Before[a]	During[b]	After[c]	Before[a]	During[b]	After[c]
All myocardial infarction	19.9	15.3	18.5	13.9	10.9	13.0	11.2	8.8	10.5
STEMI	10.5	8.3	9.7	7.6	6.1	7.1	6.2	5.0	5.8
NSTEMI	10.6	7.9	9.9	7.1	5.4	6.7	5.6	4.2	5.3
All stroke	32.2	26.3	30.4	20.1	16.3	12.3	15.7	12.7	15.2
Ischemic stroke	19.8	16.6	19.0	11.9	10.0	11.7	9.2	7.7	9.1
Hemorrhagic stroke	6.2	5.3	5.7	4.2	3.5	3.9	3.4	2.8	3.2
Transient ischemic attack	8.1	6.1	7.5	5.2	3.9	4.9	4.2	3.1	3.9

STEMI: ST segment elevation myocardial infarction; NSTEMI: non-ST segment elevation myocardial infarction
[a] before lockdown. i.e. January 1st to March 16th 2020
[b] during the lockdown. i.e. 17th March to May 10th 2020
[c] after the lockdown. i.e. May 11th to September 30th 2020

Supplementary Table S2. Interrupted time series analyses of hospitalizations for cerebro-cardiovascular events for weeks 2 to 39 of 2020 in France. with three periods: before. during and after the lockdown (weeks 12 to 19)

Hospitalization for:	Variable	Estimate	Standard Error	P-value
Myocardial infarction	Intercept	0.0047800	0.0001260	$<10^{-4}$
	Slope before lockdown	-0.0000330	0.0000203	0.1179
	Level change during/before lockdown	-0.0013700	0.0001730	$<10^{-4}$
	Slope during lockdown	0.0001690	0.0000349	$<10^{-4}$
	Level change after/during lockdown	0.0003210	0.0001640	0.0593
	Slope after lockdown	-0.0001600	0.0000293	$<10^{-4}$
STEMI	Intercept	0.0024400	0.0000713	$<10^{-4}$
	Slope before lockdown	-0.0000046	0.0000115	0.6938
	Level change during/before lockdown	-0.0006690	0.0000981	$<10^{-4}$
	Slope during lockdown	0.0000562	0.0000198	0.0077
	Level change after/during lockdown	0.0002020	0.0000929	0.0374
	Slope after lockdown	-0.0000630	0.0000166	0.0006
NSTEMI	Intercept	0.0026090	0.0000773	$<10^{-4}$
	Slope before lockdown	-0.0000280	0.0000125	0.0291
	Level change during/before lockdown	-0.0008050	0.0001060	$<10^{-4}$
	Slope during lockdown	0.0001240	0.0000214	$<10^{-4}$
	Level change after/during lockdown	0.0001240	0.0001010	0.2282
	Slope after lockdown	-0.0001070	0.0000180	$<10^{-4}$
Stroke	Intercept	0.0077020	0.0001550	$<10^{-4}$
	Slope before lockdown	-0.0000440	0.0000250	0.0864
	Level change during/before lockdown	-0.0018750	0.0002130	$<10^{-4}$
	Slope during lockdown	0.0002410	0.0000430	$<10^{-4}$
	Level change after/during lockdown	0.0002040	0.0002020	0.3185
	Slope after lockdown	-0.0002140	0.0000361	$<10^{-4}$
Hemorrhagic stroke	Intercept	0.0014730	0.0000513	$<10^{-4}$
	Slope before lockdown	-0.0000067	0.0000083	0.4269
	Level change during/before lockdown	-0.0002880	0.0000706	0.0003
	Slope during lockdown	0.0000349	0.0000142	0.0199
	Level change after/during lockdown	-0.0000140	0.0000668	0.8361
	Slope after lockdown	-0.0000310	0.0000119	0.0143
Ischemic stroke	Intercept	0.0047980	0.0000878	$<10^{-4}$
	Slope before lockdown	-0.0000410	0.0000142	0.0072
	Level change during/before lockdown	-0.0008810	0.0001210	$<10^{-4}$
	Slope during lockdown	0.0001400	0.0000244	$<10^{-4}$
	Level change after/during lockdown	0.0001350	0.0001140	0.2463
	Slope after lockdown	-0.0001040	0.0000205	$<10^{-4}$
Transient ischemic attack	Intercept	0.0019000	0.0000703	$<10^{-4}$
	Slope before lockdown	-0.0000017	0.0000113	0.8835
	Level change during/before lockdown	-0.0007680	0.0000968	$<10^{-4}$
	Slope during lockdown	0.0000845	0.0000195	0.0001
	Level change after/during lockdown	0.0000324	0.0000917	0.7263
	Slope after lockdown	-0.0000920	0.0000164	$<10^{-4}$

Supplementary Figure S3. Observed (Obs) and predicted (ITS) hospitalizations for cardiovascular events for weeks 2 to 39 of 2020 in France. interrupted time series (ITS) analysis with three periods (before. during. after the first lockdown)

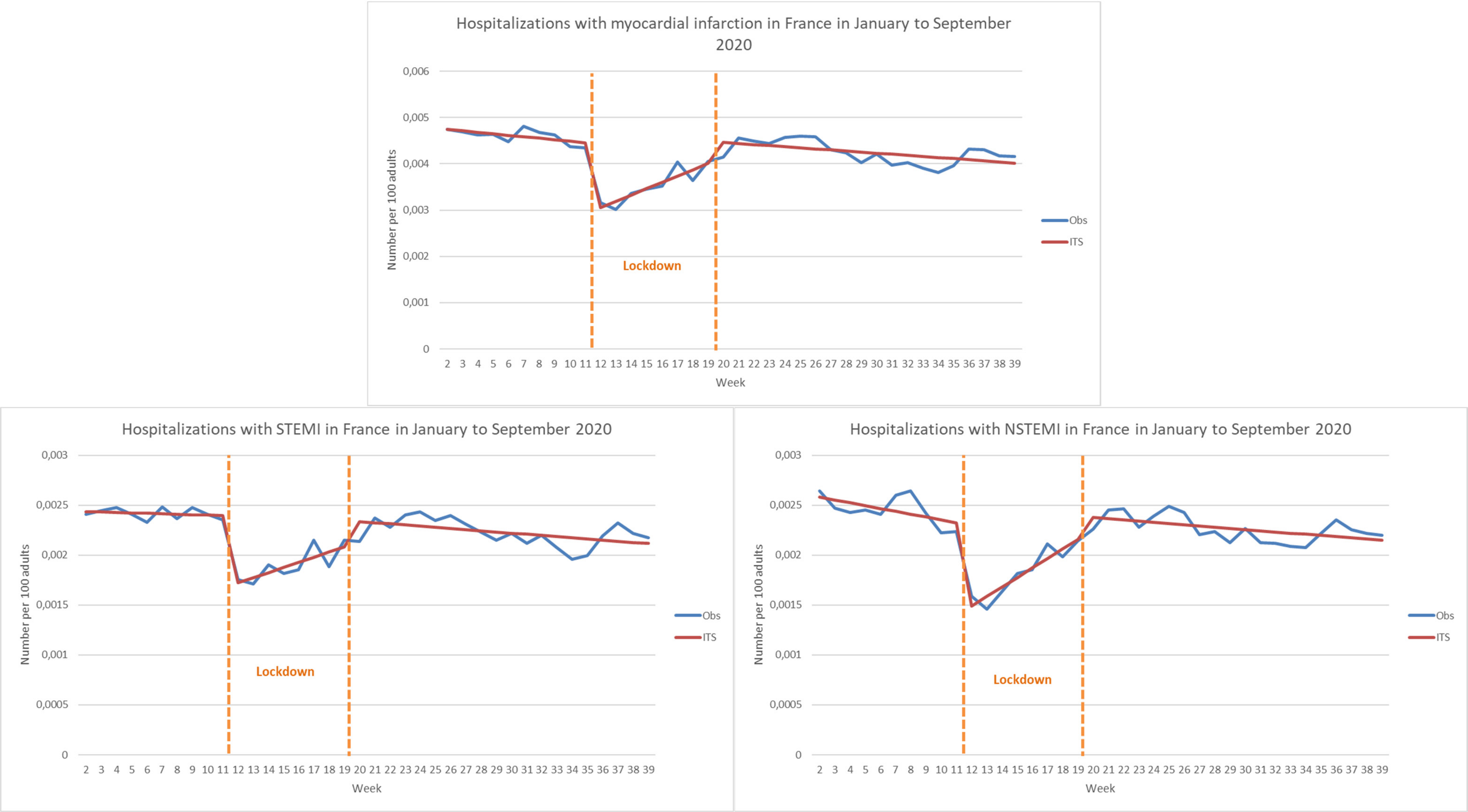

Supplementary Figure S4. Observed (Obs) and predicted (ITS) hospitalizations for cerebrovascular events for weeks 2 to 39 of 2020 in France. interrupted time series (ITS) analyses with three periods (before. during. after the first lockdown)

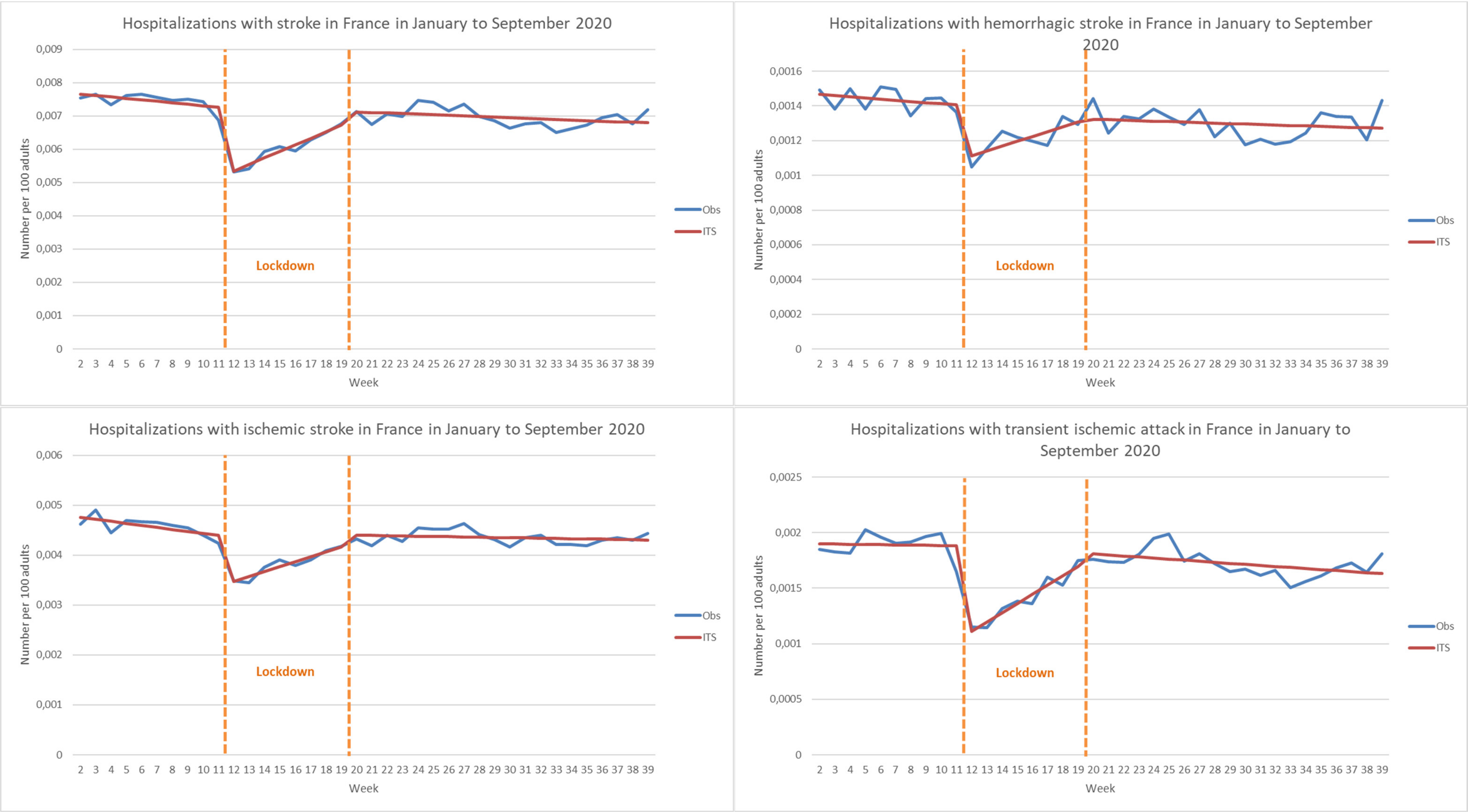

Supplement: Supplementary file 1 [file biomedicines-10-02501-s001.zip › biomedicines-1921672-supplementary.pdf]
